# A memory clinic *v.* traditional community mental health team service: comparison of costs and quality

**DOI:** 10.1192/pb.bp.113.044263

**Published:** 2015-02

**Authors:** Judy Sasha Rubinsztein, Marelna Janse van Rensburg, Zerak Al-Salihy, Deborah Girling, Louise Lafortune, Muralikrishnan Radhakrishnan, Carol Brayne

**Affiliations:** 1 Norfolk and Suffolk NHS Foundation Trust; 2 Cambridgeshire and Peterborough NHS Foundation Trust; 3 University of Cambridge; 4 King’s College London

## Abstract

**Aims and method** To compare the cost and quality of a memory-clinic-based service (MCS) with a traditional community mental health team (CMHT) service. Using a retrospective case-note review, we studied two groups, each with 33 participants. Consecutive referrals for diagnostic ‘memory’ assessments over 4 months were evaluated. Participants were evaluated for up to 6 months.

**Results** The MCS was less costly than the CMHT service but the difference was not statistically significant (mean cost for MCS was £742, mean cost for CMHT service was £807). The MCS offered more multidisciplinary and comprehensive care, including: pre- and post-diagnostic counselling, more systematic screening of blood for reversible causes of dementia, more use of structured assessment instruments in patients/carers, signposting to the third sector as well as more consistent copying of letters to patients/carers.

**Clinical implications** An MCS service offered more comprehensive and multidisciplinary service at no extra cost to secondary care.

In the UK, the national dementia strategy^1^ strongly supports the concept of timely diagnosis and intervention in dementia. The cost-effectiveness of such services has been demonstrated.^2–6^ Studies show that memory services may improve quality of life for patients and carers.^7–9^ The recognition of dementia enables the provision of safer care by considering, for example, accommodation and care needs and encouraging advanced decision-making. Reaching a diagnosis can help patients and carers to tolerate their symptoms better and suitable treatments can be considered.

Advocates of dedicated ‘memory clinic services’ (MCSs) point to the benefits of a less stigmatising setting, with a focus on psychosocial interventions, education and the promotion of research and clinical governance.^5,7,10–14^ Opponents are concerned with resources being diverted from community mental health teams (CMHTs) and point to higher costs of MCS services.^15,16^

In this study, Trust A has a dedicated memory clinic service (henceforth called MCS group) as well as a CMHT. In Trust B there is a ‘traditional’ CMHT-based service (henceforth called CMHT group) where referrals of all types are seen. We compared the costs with those of secondary care and the quality of the service offered to similar types of patients referred for a non-urgent diagnostic memory assessment in these two service models in neighbouring trusts. Patients were evaluated retrospectively using case-note review in terms of the cost and quality of care they received in secondary care for up to 6 months in similar rural/suburban areas.

## Method

### Study design

This study was a health service evaluation and used retrospective data collected routinely as part of the usual services provided in both trusts, so local research ethics committee permission was not needed.

### Demographic matching

Two rural and suburban eastern areas of England were matched using data from the Eastern Region Public Health Observatory (ERPHO). The number of people over the age of 65 years, based on general practice (GP) records in these areas, was recorded: 20 289 people in the MCS group and 21 112 people in the CMHT group. Data from the British primary care Quality and Outcomes Framework (QOF),^17^ which requires the recording of dementia and other chronic conditions, showed that the prevalence of dementia and strokes, and the recording of cholesterol and blood pressure, were similar in both groups. The Indices of Multiple Deprivation (IMD)^18^ show that the GP practices in the CMHT service (Trust B) experienced less deprivation. The range of the IMD was 11.5–17.7 for the MCS and 5.7–8.96 for the CMHT service.

### Patient identification

We examined all referrals received over the time frame of the study (4 months) from specific, predetermined GP practices in these two rural/suburban areas. All patients included in the study had to fulfil Department of Health memory clinic referral criteria.^19^ These criteria state that memory services will see all patients with subjective memory problems or change in everyday function, or a carer’s report of change in a patient over a period of more than 6 months. Patients referred should have no previous or definitive diagnosis of dementia. The Department of Health stipulates that memory services will not see patients where urgent treatment is needed, for example those with more complex behavioural and psychological problems, patients with suicidal ideation, psychotic behaviour or a crisis situation from the carer’s perspective. Patients with early-onset dementia (age under 64 years) were also excluded because the two areas have different referral pathways for such patients. The MCS service received two referrals of early-onset dementia, which were excluded from this study; there were no such referrals in the CMHT service. All referrals had to have been received between August and November 2011 in both services.

For the MCS group, a computerised search of all memory clinic patients referred by GP practices in the designated area of the trust was performed for the specified time period. Thirty-four consecutive patients were identified and all fulfilled the Department of Health memory clinic criteria.^19^ One patient refused the diagnostic interview after pre-diagnostic counselling and that patient was excluded. Occasionally, patients may be passed on to the crisis team or CMHT, but none on our list had been dealt with in this way.

For the CMHT group, a member of the research team (M.J.V.R.) manually searched all consecutive referrals to the service from particular GP practices from a central written database. Thirty-three referrals were included in the CMHT group (by coincidence, the same number of patients as the MCS group). These 33 patients all fulfilled Department of Health memory clinic criteria^19^ and were identified by consecutively recruiting from the original list. Patients were excluded only if they did not fulfil memory clinic criteria.^19^ A second member of the research team (J.S.R.) ensured that all referrals met these criteria for inclusion or exclusion. There was uncertainty as to whether to include one patient and a senior nurse’s opinion from the MCS team was sought to arbitrate.

### Questionnaires

#### Quality of service

A data extraction sheet to assess the quality of the service was developed for this study. It was used for case-note analysis and captured information routinely collected by clinicians for assessment purposes (Box 1). The quality criteria were chosen based on the National Institute for Health and Care Excellence (NICE) guidance for dementia services,^20^ the Memory Services National Accreditation Programme (MSNAP) criteria where they could be applied to both services,^21^ and literature evidence.^10,11,22^ We pragmatically assessed whether the data could be extracted retrospectively from the services. This information was obtained from computerised and handwritten notes from the initial contact with the patient and for the following 6 months of contact with the mental health service. The psychiatrists involved in the data collection conferred about any uncertainties with recording of data. M.J.V.R. and Z.A.-S. each initially screened at least four sets of case records together with J.S.R. to ensure interrater reliability in recording of data. All entries were scrutinised by J.S.R. to ensure data entry was consistent. Any missing information was noted. It is the view of clinicians in both trusts that GPs are best suited to conduct physical examinations and these are stated in the requirements for referral to the service by the MCS trust. However, individual psychiatrists often choose to conduct some aspects of the physical examination themselves and the extent to which clinicians were doing this was noted.

#### Cost of service

The Client Service Receipt Inventory (CSRI)^23^ was adapted for this study (available from the authors on request). This includes all the mental health service costs for each individual patient from the initial point of contact and then all subsequent contacts with the mental health service over the following 6 months. Costing stopped at the point the patient was discharged back to the GP. However, patients found to need more extensive follow-up for more severe or complex behavioural and psychological symptoms of dementia (BPSD) were referred to the CMHT service in Trust A (one patient) and Trust B (three patients). Costs were excluded from that point. Costing was calculated from the perspective of National Health Service care^24^ (secondary care only evaluated in this study) rather than from a wider medical or societal perspective. Costs for hourly contact with professionals were mainly derived from the unit costs for health and Social Services compendium and included ‘on costs’, for example for a consultant psychiatrist this includes salary, national insurance and superannuation, qualifications, overheads, ongoing training and capital overheads.^25^ From this document,^25^ the cost per hour for consultant time is £162 (including on costs such as administrative support and buildings) and the cost per hour for a non-medical clinician (e.g. CMHT nurse) is £44. The costs of drugs prescribed by secondary care were derived from the *British National Formulary*.^26^ The costs of scans were derived on the basis of the Department of Health’s Dementia Commissioning Pack.^27^ Costs incurred as a result of time spent on discussion and meetings were based on the size of individual teams, allowing for an average of 6 min discussion per patient (team sizes and calculations available from the author). The average time taken for domiciliary

**Table 1:** Demographic data and results of assessment in memory clinic service (MCS) group and community mental health team (CMHT) group

	MCS group (*n* = 33)^a^	CMHT group (*n* = 33)^a^	*P*	Comments
Mean age, years	80	84	0.03	Significantly older in Trust B
				
Mean age when leaving school, years	15	15	/>0.05	Not recorded in 4 patients in MCS and in 20 patients in CMHT
				
Female, *n* (%)	19 (58)	22 (67)	/>0.05	
				
MMSE, median	23.5	25	0.2	*n* = 32 in both groups
				
ACE-R, median (range)	67 (76)			CMHT group not analysed as only 9 patients had ACE-R done, *n* = 31 in MCS group
				
Accommodation – independent/ sheltered, *n* (%)	32 (97)	30 (94)	/>0.05	
				
Seen with relative/friend/carer, *n* (%)	33 (100)	24 (73)	<0.001	Clinicians in CMHT group may have contacted relative by telephone after interview
				
Mean days to be seen (s.e.), *n* (%)	25 (3)	20 (3)	0.23	
				
Mean months since symptom onset	23	24	0.77	12 not known in CMHT group
				
Received pre-diagnostic counselling, *n* (%)	32 (94)	2 (6)	<0.0001	
				
Dementia blood screen examined, *n* (%)	33 (100)	24 (73)	0.001	
				
Physical exam done by GP/psychiatrist, *n* (%)	16 (48)	14 (42)	n/s	
				
Functioning examined formally, *n* (%)	24 (73)	1 (3)	<0.0001	e.g. Bristol Activities of Daily Living^30^
				
Behaviour examined formally, *n* (%)	22 (67)	1 (3)	<0.0001	e.g. Cambridge Behavioural Inventory^31^
				
Global assessment formal, *n* (%)	33 (100)	14 (42)	<0.0001	e.g. HoNOS,^32^ EQ-5D-5L^33^
				
Depression examined formally, *n* (%)	2 (6)	1 (3)	>0.05	e.g. Geriatric Depression Scale^34^
				
Risk assessment, *n* (%)	31 (94)	22 (67)	0.02	
				
Patient/carer sent copy of GP letter, *n* (%)	29 (88)	14 (42)	<0.0001	

ACE-R, Addenbrooke’s Cognitive Examination–Revised; GP, general practitioner; HoNOS, Health of the Nation Outcome Scales; MMSE, mini-Mental State Examination; n/s, non-significant.

a. Unless otherwise stated.

visit and/or administrative time costs were calculated on the basis of discussions with representatives from professional groups in each of the teams or on data recorded by team members (average times for appointment available from the author on request). The mileage travelled by clinicians was calculated using the Automobile Association Website (www.theaa.com). Travel and transport costs are part of general overheads in the unit costs of health and social care,^25^ but as this was anticipated to be an area of difference between the two models, this was calculated separately for each patient seen at the rate of 54p/mile up to 3500 miles as suggested in this unit cost document.

**Box 1** Measures extracted regarding the quality of the memory service

- Background characteristics (age, gender, marital status, employment, school-leaving age, accommodation)
- Waiting time to be seen
- Symptom time prior to referral
- Presence of a carer, relative, friend
- Pre-diagnostic counselling
- Dementia blood screen: ordered, examined
- Informal assessment of functioning, behaviour, depression, global assessment
- Structured questionnaires to assess functioning, behaviour, global assessment
- Brain imaging
- Physical examination (record from GP/psychiatrist)
- MMSE, ACE-R or other cognitive tools utilised
- Record of a diagnosis
- Record of risk assessment
- Record of post-diagnostic advice to patient/carer
- Record of discussion about driving
- Copying of letters to patients/carers

ACE-R, Addenbrooke’s Cognitive Examination-Revised; GP, general practitioner; MMSE, mini-Mental State Examination.

#### Analysis

Data were analysed using Excel 2007 and Stata Version 12.1 for Windows (χ^2^, Fisher’s exact tests if less than 5 in a cell, Wilcoxon rank sum test). Parametric and non-parametric tests were applied, as appropriate, to evaluate costs and quality of care provided. To be conservative and because non-parametric distributions were predicted, the cost data were analysed using the Wilcoxon rank sum test.

## Results

### Quality data

A similar range of diagnoses were seen in both areas (Alzheimer’s or mixed dementia: 17 in MCS group, 15 in CMHT group; vascular dementia: 9 in MCS and 8 in CMHT; Lewy body dementia: 0 in MCS group, 2 in CMHT group; mild cognitive impairment: 6 in both groups; depression: 0 in MCS and 1 in CMHT; other diagnoses: 1 chronic subdural in MCS group and 1 multiple sclerosis-related cognitive impairment in CMHT group). Demographic data and the analysis of data collected during patient assessments are shown in Table 1.

Diagnostic assessments included a clinical assessment of behaviour, functioning and a global assessment of severity in nearly all patients, with no significant differences between groups on these variables. Some structured questionnaires (e.g. EQ-5D,^33^ Cambridge Behavioural Inventory^31^) were sent to patients and carers before the actual appointment in the MCS group. Others were administered by clinicians during the clinic appointment. However, structured assessments in patients and carers using symptom rating scales in these domains were not routinely done in the CMHT group. A Mini-Mental State Examination (MMSE)^28^ was performed in all patients except one in each group. In the MCS group, the Addenbrooke’s Cognitive Examination-Revised (ACE-R) test^29^ was performed routinely, with a median score of 67, but it was not performed routinely in the CMHT group. Computed tomography head scans were ordered as part of the assessment to a similar extent in both groups (19 in MCS and 17 in CMHT), whereas scans that had been done previously and considered by the clinician to be recent enough to be helpful amounted to a further 6 in the MCS and 5 in the CMHT group. So, only 8 MCS (24%) and 11 CMHT (33%) patients did not have scans available for diagnostic purposes. Scanning is widely available in both trusts and it was patient preference and some clinician guidance that determined whether a patient had a scan or not. Diagnoses were recorded by clinicians in 100% of letters sent to GPs. A psychologist saw two patients in the MCS group (one for neuropsychological testing and one for cognitive stimulation therapy) and two patients in the CMHT group had further neuropsychological testing.

The post-diagnostic advice given by clinicians to patients and/or carers from the MCS *v*. CMHT group in percentage terms was signposting: to the third sector (70% *v*. 24%; *P* = 0.0002); for welfare benefits (55% *v*. 36%; *P*>0.05); to Social Services (67% *v*. 48%; *P*>0.05); advanced

**Table 2:** Mean costs in memory clinic service (MCS) group and community mental health team (CMHT) group^a^

Costs	MCS group £ (mean per person ±s.d.)	CMHT group £ (mean per person ±s.d.)
Total Costs	742 (250)	807 (375)
		
Direct costs	271 (82)	252 (124)
		
Office costs	182 (81)	224 (130)
		
**Travel time**^b^	**76 (59)**	**186 (106)**
		
Mileage costs	28 (21)	32 (31)
		
Multidisciplinary team costs	44 (15)	30 (41)
		
Scan costs	74 (67)	69 (72)
		
Drug costs	67 (103)	12 (31)

Bold denotes significance.

a. Small differences in the total means can be accounted for by rounding off.

b. The only significant difference between the groups was in the travel time costs *P* < 0.0001; *z* = –5.14 (Wilcoxon rank sum test). The drug, scan and multidisciplinary team costs were not formally analysed as their distributions were not suitable for parametric testing and there were too many ties for the Wilcoxon rank sum test.

planning discussions (55% *v*. 45%; *P*>0.05). Interviewers were better at documenting whether patients were drivers in the MCS group (information not recorded in three patients in the MCS group and nine patients in the CMHT group). Anti-dementia drugs were prescribed in 65% of patients eligible for prescriptions (if patients had Alzheimer’s disease, mixed dementias or Lewy body dementia and MMSE scores greater than 10). Payment methods differed between groups, with the CMHT passing on prescribing to GPs after 1–2 months compared with the MCS group, where clinicians continued to prescribe for 3–4 months.

### Patient pathways

The major significant difference was that just under half of patients in the CMHT group (*n* = 15, 45%) were seen only by a doctor at their usual place of residence and then discharged, whereas in the MCS group 97% were seen by both a doctor and a non-medical clinician (once or twice). In the CMHT group, 55% of patients were seen by non-medical clinicians several times (range 2–11). Although some patients received no follow-up in the CMHT group, others received extensive follow-up within the 6-month period, incurring increased travel time, mileage and face-to-face costs. Most medical input was from the clinic base for the MCS group, but 85% of patients also received a domiciliary visit by a non-medical clinician either pre- or post-diagnosis (or both).

### Costs of service

The total costs per person to secondary care between the MCS and the CMHT groups over 6 months were not significantly different in the non-adjusted analysis or the analysis adjusted for age and MMSE score. The mean total cost of care in the MCS group was £742 (median £722) and in the CMHT group it was £807 (median £833). Travel costs were significantly higher in the CMHT group where all patients were seen at their usual place of residence (*Z* = –5.14, *P*<0.0001, effect size –0.63). Consultants travelling to see patients would often add a cost of £162/hour to each assessment in the CMHT group. This expense was not needed for clinic-based assessments by consultants in the MCS group. The costs for non-medical staff to travel to see patients more frequently than in the MCS group also added to this overall travel cost figure. Other costs between groups were not significantly different using non-parametric analysis (Table 2).

## Discussion

This paper highlights the cost and quality differences between two service models for patients referred with concerns about their memory. The total costs to secondary care were less with the MCS-based service than the CMHT-based service (median cost of £722 *v*. £833 per patient), but this difference was not statistically significant. The MCS offered significantly more multidisciplinary care to a greater number of patients than the CMHT service. Both services offered a high-quality diagnostic service but we argue that the MCS service was able to offer more systematic and comprehensive care, including pre-diagnostic counselling, more systematic screening of blood tests for reversible causes of dementia/comorbidity, more extended cognitive examination and structured assessment tools, better evidence of signposting to the third sector as well as copying of letters to patients and carers. It has been demonstrated that there is greater satisfaction with multidisciplinary assessment^35,36^ where diagnostic and management options are explained to both patient and caregiver.

It is possible that patients in the CMHT group were not typical for an MCS or that a selection bias was introduced, with only 33 patients in the CMHT group. However, we feel this is unlikely as the Department of Health memory clinic criteria were applied to all referrals accepted into the study in a systematic way.

We acknowledge that the numbers included in the study were small and the findings can only be regarded as preliminary. However, we cannot exclude the possibility that a CMHT service may be more economical for all types of patients as it was beyond the scope of this study to examine the costs for all patients entering CMHT services in both areas. The study was also not a full economic evaluation where costs and outcome data (such as delays to institutional care) are combined to reach conclusions. The CSRI^23^ as adapted for this study only examines costs to secondary mental healthcare and not primary care, social care or carer time costs. Using the CSRI, we detailed the patient’s involvement with doctors and other clinicians as accurately and comprehensively as possible. In real life, clinicians do not return to base between patient visits so costs may have been inflated in both services for travel time. We were aware that there seemed to be differences between groups in the rate whereby prescribing was handed over to GPs.

This was a retrospective service evaluation and we encountered many of the pitfalls of examining data that were not specifically collected for research purposes. However, the pragmatic design of this study also means it is more reflective of actual practice and therefore less subject to a Hawthorne effect.

Stakeholder views had been sought in both trusts and satisfaction was high with both services in the year of the study, but this was not evaluated specifically in this research and satisfaction cannot be inferred from these data.

There will, of course, be differences among clinicians about what determines the quality of a memory service and we acknowledge our own subjectivity. However, we took a pragmatic view on which variables to include, based on the literature and the information we were likely to be able to obtain from retrospective data in these two services. Other quality indicators for a memory service may be helpful to consider in future studies, for example the rate of reversible causes found, the rate of ‘no diagnosis’ made, the range of diagnoses or the rate at which drugs were accepted by eligible patients. However, this sample was too small to find significant between-group differences on these indicators.

The IMD in the MCS group was lower than in the CMHT group. This could possibly influence referral patterns but we acknowledge that this is a complex issue, involving the attitudes of patients, families and their referring GPs. Ethnicity was not specifically matched for in this study and this is acknowledged as a study limitation. We acknowledge that both groups had higher than expected rates of patients not receiving anti-dementia drugs. Clinicians did not always offer the drug, because they were concerned patients would not comply with taking the medication. However, some patients refused the drugs because of possible side-effects or other factors.

Another emerging care model in the UK utilises the services of ‘allied mental health professionals’ in making diagnoses and offering interventions with medical input not provided face to face for most patients.^5,37,38^ It may be argued that some of the diagnostic quality provided by a ‘medical’ view on diagnosis may be compromised in such services and this needs further evaluation.
